# Novel Use of Video Logs to Deliver Educational Interventions to Black Women for Disease Prevention

**DOI:** 10.5811/westjem.2021.12.54012

**Published:** 2022-02-28

**Authors:** Mandy J. Hill, Sandra Coker

**Affiliations:** * McGovern Medical School and University of Texas Health Science Center at Houston, Department of Emergency Medicine, Houston, Texas; † The University of Chicago Pritzker College of Medicine, Department of Emergency Medicine, Chicago, Illinois

## Abstract

**Introduction:**

Cisgender Black women comprise 67% of new human immunodeficiency virus (HIV) diagnoses among women in the South and are 11 times more likely to become HIV positive than White women in Texas. Optimal progress toward ending the HIV epidemic requires strategies that will interrupt transmission pathways in hotspot locations like Harris County, TX. Researchers are calling for public health interventions that can prevent HIV and sexually transmitted infections (STI) transmission; thus, we launched the first video log (vlog)-based, pilot HIV prevention intervention.

**Methods:**

In a prospective. randomized controlled trial of two educational intervention strategies delivered as vlogs eligible participants were randomized to either 1) an interactive gaming, education-based strategy, or 2) a storytelling, education-based strategy. Eligible participants were cisgender Black women being seen in the emergency department (ED) for a non-emergent condition who reported recent condomless heterosexual sex, were ages 18–45, and had social media access. Enrolled women completed a screening assessment, informed consent, randomization, and 10-item pre-and-post assessments with true/false statements before and after viewing a brief vlog on a tablet device to identify changes in knowledge before and after being educated on HIV/STI transmission.

**Results:**

Twenty-six women were randomized to the Taboo group, an interactive gaming, education-based strategy, (14 [53.8%]), or to storytelling, an education-based strategy using non-fictional and fictional case scenarios (12 [46.2%]). Taboo participants self-identified as African-American (12 [85.7%]), Black (1 [7.1%]) or “other” (1 [7.1%]), were younger (28.6% were ≥ 30 years), single (57.1%), reported a previous STI (8 [57.1%]), and were likely employed (57.2%). Storytelling participants self-identified as African-American (7 [58.3%]) or Black (5 [41.7%]), were older (49.9% were ≥ 30 years), in a relationship but not married (50%), and half were unemployed. Highest level of education and monthly income varied. The storytelling strategy increased knowledge in two areas and the Taboo strategy increased knowledge in one. No intervention effect was identified in three areas, and a significant decrease in knowledge (P < .0001) was discerned in eight areas for Taboo and six areas for storytelling.

**Conclusion:**

Further research is necessary to confirm whether delivery of HIV prevention interventions with vlogs is a useful approach for HIV-vulnerable populations. Findings suggest that vlogs are a feasible approach to brief behavioral interventions during an ED visit.

## INTRODUCTION

New human immunodeficiency virus (HIV) and sexually transmitted infections (STI) are major public health problems for cisgender Black women who account for 67% of new HIV diagnoses among women in the South and STIs at a much higher rate than other women.^1^–^3^ The emergency department (ED) is a usual source of care for many Black women who are more likely to use the ED for primary care than others.^4^,^5^ The ED visit provides an opportunity to engage Black women in HIV prevention. Prevention interventions for HIV have leveraged technology to facilitate education to vulnerable populations. Wingood et al led randomized controlled trials (RCT) of behavioral interventions with computer-delivered interventions for Black women to motivate behavior change with individuals and groups.^6^,^7^,^8^ These interventions demonstrated efficacy with Black women; however, we need innovation to translate intervention efficacy through web-based delivery. Bond et al (2019) led an HIV prevention intervention for Black women using electronic health (eHealth) videos to offer education through culturally centered, entertainment-education health messages to increase awareness of HIV prevention methods.^9^

Our goal was to add innovation to eHealth videos with video logs (vlog). Within the last five years, researchers began to explore the utility of vlogs as a means of engaging patients,^10^ testing social network interventions, and promoting healthy behavior changes.^11^ Vlogs are brief videos that are developed by users and shared online through social media platforms.^11^,^12^ Vlogs can connect peers to one another through observation of others’ behavior.^11^,^13^ Evidence affirms vlogs as a primary source of information for many Black women. Using vlogs to deliver interventions is still novel, and to our knowledge has only been used for health promotion in one other study (to increase physical activity).^11^ Adapting vlogs for health education through vehicles that Black women are already using for information is a necessary addition to HIV prevention science.

This “Debunking Myths” pilot study is our first attempt at enhancing the sexual health knowledge of Black women during an ED visit. We chose myths regarding transmission pathways that were discovered through a thematic content analysis of interview transcripts of Black women who described their sexual experiences, norms, and practices.^14^–^16^ Due to the prevalence of myths conveyed during the interviews, we designed this pilot study that uses vlogging to educate Black women on HIV/STI transmission to protect their own sexual health. The goal was for vlogs to resonate with Black women and inform healthy sexual decision-making. We hypothesized that storytelling would be more effective because it was more culturally relevant for Black women.

## METHODS

### Study Design

This was a prospective, comparative effectiveness RCT whereby eligible participants were recruited during an ED visit at two hospital sites. Enrolled participants were randomized (2019–2021) to either 1) an interactive gaming, education-based strategy, or 2) a storytelling, education-based strategy. Both strategies displayed a vlog on a tablet device. The primary outcome was an increase in knowledge regarding how HIV and STIs are transmitted. This study was reviewed and approved by the UTHealth Center for the Protection of Human Subjects (HSC-MS-19-0488).

**Population Health Research Capsule**

What do we already know about this issue?

*Cisgender Black women are at risk for human imunodeficiency virus (HIV) and sexually transmitted infections (STI), which warrants new prevention strategies capable of disrupting transmission patterns.*

What was the research question?

*Would video logs (vlog) delivered as storytelling be more effective at increasing knowledge than a question-and-answer model?*

What was the major finding of the study?

*Although innovative, the vlog approach did not increase knowledge on HIV and STI transmission as we hypothesized.*

How does this improve population health?

*Vlogging can connect with diverse audiences in a way that aligns with societal and community-based communication norms to motivate behavior change.*

### Enrollment Process

Cisgender Black women were screened during an ED visit for a non-emergent condition (an acuity level of three or higher on the Emergency Severity Index scale). We recruited those who met the inclusion criteria based on the electronic health record (EHR) screening at two participating hospitals, a public hospital with a Level III trauma designation (volume: 90,000 ED visits/year) and a private hospital with a Level I trauma designation (volume: 70,0000 ED visits/year) **(**Table 1).

The entire research process, including in-person recruitment, eligibility assessment, enrollment and informed consent, randomization, pre-test, watching the vlog, and the post-test took place within 20 minutes for each participant enrolled. The Taboo vlog^17^ was eight minutes and 46 seconds long. The storytelling vlog was 11 minutes and 11 seconds long. Research participants viewed the vlog during wait times of an ED visit, after they were placed in a patient room following triage and prior to discharge, transfer, or escalation of care beyond the ED.

### Randomization

After we obtained consent for study participation, each woman was assigned a study identification number that linked her to study data. When a new participant was enrolled and pre-screened, the randomization button in REDCap hosted at UTHealth Science Center at Houston was selected to assign the participant to a study arm.^18^,^19^ Randomized assignments were placed in an Excel^20^ file (Microsoft Corporation, Redmond, WA) and uploaded to REDCap online software (REDCap Technologies, LLC, Fort Lauderdale, FL)^18^,^19^ by a statistician.

### Study Assessments

Participants were screened using REDCap software^18^,^19^ accessed on a tablet device that assessed demographic, structural, environmental, and behavioral factors. Once deemed eligible and consented, participants were randomized.

#### Pre-test

Participants completed a pre-test using Qualtrics software (Qualtrics XM, Provo, UT).^21^ The pre-test assessed baseline knowledge on HIV/STI transmission routes through presentation of 10 myths and facts as single sentences with a true/false response format.

#### Post-test

Following vlogs, participants completed a post-test, which was identical to the pre-test, to evaluate whether the education intervention had an effect.

### Intervention Strategies

Each intervention used a distinct educational approach whereby misinformation on how HIV/STIs are transmitted was communicated first. Then, accurate information was shared in a way to correct the original misinformation.

**Image 1:**
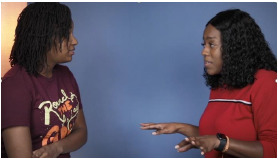
A visual of the Taboo intervention strategy depicting communication between a healthcare physician and a Black woman.

Taboo is an interactive gaming, education-based strategy. The vlogger is dressed in casual clothes and presents information as facts followed by a surprise graphic interchange format or unexpected buzzer, which serves as a signal of misinformation. Following the sound of the buzzer, the vlogger appears with a white coat and stethoscope in the adjacent lamp shade to share accurate and factual information **(Image 1)**.

**Image 2:**
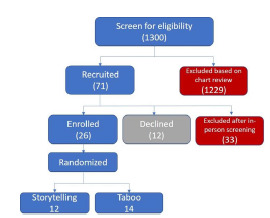
A visual of the storytelling intervention strategy depicting a conversation between two Black women. Note: Image reflects actor portrayals illustrating video log communication.

Storytelling is an education-based strategy using non-fictional and fictional case scenarios. Storytelling involved two vloggers having a conversation in a social setting as friends **(Image 2)**. Vloggers recount scenarios in a casual manner with use of modern colloquialisms that are common to Black women. Myths regarding HIV/STI transmission were presented and refuted. This entertaining yet relatable education strategy aligned with the study aim to address a specific demographic with a culturally relevant approach.

### Sample Size

**Figure 1:**
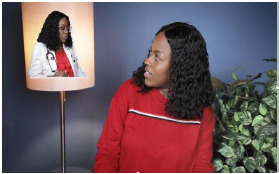
CONSORT* flow diagram of the participants. *Consolidated Standards of Reporting Trials.

We enrolled 26 women (14 in Taboo, 12 in storytelling). Our sample size was based on reports that 20 participants would be sufficient to determine salient beliefs.^22^–^24^ Thus, a sample size of 26 (Figure 1) was deemed sufficient to discern changes in knowledge.

### Data Analytic Plan

We analyzed quantitative results from the assessments using SPSS statistical software (IBM Corporation, Armonk, NY).^25^ The file was split by randomization group to compare the intervention effect on knowledge and discern demographic differences between participants in each group. We assessed knowledge based on responses to true/false statements using frequency analyses and Pearson’s chi-squared tests with *P*-values. Incomplete data was identified and is reflected in the study findings (see Tables).

## RESULTS

### Demographics

Participants were asked how they self-identify by race **(**Table 2). Taboo participants (85.7%) were more likely to self-identify as African-American than women randomized to storytelling (58.3%). One woman randomized to Taboo described her race as “other.” Women randomized to Taboo were younger: 50% (7/14) were 20–24 years of age. The age differences between groups were statistically significant (*P* = 0.05). Half of participants in storytelling were single (41.7%) or in a relationship but not married (50.0%). Most women in Taboo were single (57.1%) or in a relationship, but not married (28.6%). In the storytelling cohort, the age range of the women was broader: 16.7% were 18–19 years; 25% were 25–29 years; and 33.3% were 35–40 years. Education level varied with 35.7% of participants reporting that they had completed secondary education. Most participants (57.2%) in the Taboo arm were employed either full-time or part-time. We observed variance in household income where 42.9% of participants reported a monthly income ≤ $1,000 and 35.7% reported a monthly income **≥** $2,001. Half of participants in the storytelling cohort were unemployed and reported a household income of $501–$1,500. The differences noted between groups in all categories, except for age, were not statistically significant **(**Table 2).

### Reported Behaviors

Behavioral characteristics of the women in the study sample were based on sexual experience, history of STIs, access to social media, and condomless sexual activity (Table 3). Heterosexual, condomless encounter within the prior three months was reported by 92.9% of women randomized to the Taboo study arm and 91.7% of those randomized to storytelling. Most women reported knowledge of their current HIV status as negative (78.3% in Taboo, and 91.7% in storytelling). Most women in the Taboo group (57.1%) reported a history of STI, while most women randomized to storytelling did not have a history of a STI (66.7%). All participants reported access to social media. There were no significant differences between groups regarding reported behaviors **(**Table 3).

### Assessment of Intervention’s Initial Efficacy

Findings of the Taboo and storytelling interventions demonstrate that these educational strategies have preliminary efficacy at influencing perceived knowledge on how HIV/STIs are transmitted among Black women who were exposed to the intervention. The directionality of that impact revealed variance across three responses: increased knowledge; no intervention effect; or decreased knowledge (Table 4).

### Increased Knowledge

The Taboo educational strategy showed preliminary effectiveness at reinforcing knowledge in area one **(**Table 4). One participant in this study arm responded incorrectly during the true/false statement during the pre-test but responded correctly after the intervention. Conversely, the storytelling strategy increased knowledge in areas two and three **(**Table 4). Knowledge was retained before and after the intervention among participants who responded correctly during the pre-test. Knowledge increased for one participant who responded incorrectly in area two and increased for two participants in area three. Only one participant responded incorrectly in area three during the post-test.

### No Intervention Effect

The Taboo educational strategy elicited no change in knowledge before and after the intervention in areas 2–4, nor did storytelling elicit a change in knowledge (Table 4).

### Decreased knowledge

There was a decrease in knowledge before and after the intervention among 11 true/false statements in both study arms. In contrast to the increase in knowledge described above in Taboo, knowledge regarding area 1 decreased before and after the intervention for two participants who were randomized to the storytelling study arm. There was no change in knowledge among 10 participants who responded correctly prior to storytelling. The decrease in knowledge in the Taboo cohort was significant (P < .0001) in areas 5–12 (Table 4). A significant decrease in knowledge regarding the ability to prevent HIV transmission with appropriate medications among people who are HIV positive was noted (*P* =.006). Although not significant, a decrease in knowledge before and after the intervention was also identified in areas 13 and 14 (Table 4).

Among 12 participants randomized to the storytelling cohort, the decrease in knowledge before and after the intervention met a *P*-value of .00 in six areas. Those six areas match areas 7–12 described above in reference to Taboo. The decrease in knowledge in area 5 had a *P*-value of .001 and a *P*-value of .004 in area 6 (Table 4).

The findings summarized in the table suggest that both interventions had a comparable effect on decreasing HIV/STI knowledge of participants despite any demographic differences noted. Similarly, areas where a decrease in knowledge was noted (but without significance) applied to both interventions relative to knowledge in area 13 and 14. However, significance in the change of knowledge before and after the Taboo educational intervention regarding area 15 was not found in storytelling (*P* = .02).

## DISCUSSION

Study findings contribute to the growing body of literature affirming the feasibility of integrating brief interventions within an ED visit.^4^ The ED is an ideal clinical environment for brief interventions that promote sexual health because it offers flexibility during wait times for nonemergent conditions to engage at-risk populations and link patients to primary prevention strategies that promote health.^14^,^15^,^26^–^34^ We pilot-tested an intervention approach in the ED using vlogging to deliver a brief educational strategy to Black women aimed at increasing their knowledge of how HIV/STIs are transmitted. Pilot study findings support a future prevention intervention aimed at promoting sexual health. To our knowledge, this is the first study to pilot-test vlogging as an educational intervention strategy for HIV/STI prevention.

Changes in knowledge by vlog educational strategy varied. Storytelling was more effective at increasing knowledge than Taboo. Similarly, a study among Black youth in Nigeria found that an educational digital storytelling intervention (EDSI) increased their perception and knowledge of HIV risk.^34^ In our study, women randomized to the storytelling cohort learned or experienced reinforcement of their current knowledge on HIV/ STI transmission in relation to bodily fluids, specifically semen and urine. Women randomized to the Taboo cohort learned or experienced reinforcement of their current knowledge that birth control cannot prevent STIs. When knowledge increased in one intervention, there was either a knowledge decrease or no effect in the comparable strategy.

The variance in vlogs’ efficacy in increasing HIV/STI knowledge of transmission routes compared to other education strategies could have been influenced by differences in study design. Computer-based interventions and peer education interventions are generally found to be effective at improving HIV-related knowledge.^36^ All studies included in a meta-analysis with a peer education component showed a significant increase in knowledge of HIV/STI transmission in the intervention group.^36^–^39^ The EDSI used a RCT design that involved 16 intervention sessions with an eight-week follow-up period.^34^ Sakha et al (2013) conducted a quasi-experimental study using a multimodal intervention strategy and assessed intervention effects over two months.^40^ This strategy was effective at increasing general knowledge about HIV/STIs among the 80 women enrolled (*P* <0.001). As in our study, Sakha et al found that their health education intervention increased participants’ knowledge in different aspects.^40^ Another multimodal strategy that was used as an educational intervention and included a one-hour educational session demonstrated a significant increase in knowledge (28 vs 21; *P* <.001).^41^ Our “Debunking Myths” study also used a RCT of two interventions with no control group and took place within one brief session; thus, temporality of intervention effect could not be assessed.

There were also differences in baseline knowledge among the studies. Findings of the pre-test with the EDSI revealed that participants had low HIV knowledge.^35^ Conversely, the pre-test in the “Debunking Myths” study revealed that participants had a high level of HIV knowledge during the pre-test. There was a significant decrease in knowledge among participants between the pre- and post-test. This finding contradicts previous research^42^ illustrating the effectiveness of peer education and storytelling strategies at increasing HIV knowledge.

Taboo participants were confident and steadfast in their HIV/STI knowledge before and after the intervention regarding knowledge that ejaculation was not required for STI transmission or urination did not prevent STI transmission. Storytelling was effective at increasing knowledge in these areas. Neither Taboo nor storytelling had an effect on participants’ knowledge that their own monogamy was not effective at preventing STI transmission. This sustained knowledge suggests that the Black women enrolled in the study were aware that they were at risk for HIV/STIs despite their own monogamy. This awareness is a very important finding as lack of sexual risk awareness among Black women has been a consistent finding in the literature.^43^–^47^ Within this sample, some Black women had an accurate perception of their risk for contracting HIV/STI risks. Neither vlog intervention strategy, Taboo or storytelling, had an impact on their confidence in that knowledge.

We hypothesized that storytelling would be superior to Taboo at increasing knowledge, but we did not expect that knowledge would decrease. Although most participants demonstrated significant HIV/STI knowledge during the pre-test, after reviewing the vlog they changed their responses. This change indicates that the vlog intervention either decreased their knowledge or decreased their confidence in their initial response prior to vlog exposure. While the humor and cultural competency of the vlog strategy was well received by many participants, as evidenced by their laughter and verbal engagement while viewing the vlog, the casual communication style could have diluted the potential for intervention efficacy. Findings did not offer compelling evidence that vlogs increased knowledge of HIV/STIs or that they could debunk myths regarding how HIV and STIs are transmitted. Additional research is needed to identify best approaches to leverage vlogs as a health communication medium to effectively increase knowledge on HIV/STI prevention among HIV/STI vulnerable populations.

## LIMITATIONS

The small sample size made it challenging to discern significant differences between the two groups before and after the intervention. This is the first time to our knowledge that a vlog was used to offer education on HIV/STI prevention, and the communication strategy we used may have prioritized cultural competency over message clarity. Additionally, there was a significant change in our staff, requiring re-training and interruptions with enrollment. We paused enrollment for 10 months during the COVID-19 pandemic to minimize risk to the research staff and the patient population. As the vast majority of research on HIV prevention educational strategies is conducted outside the US, those findings may be less applicable due to differences in culture and/or communication norms.

We should also point out that although the randomization was established by a statistician and set up with the randomization model within REDCap,^18^,^19^ the randomization did not appear adequate given the differences in demographics between groups. Given the unbalanced sample between groups, selection bias may have occurred and led to difficulty with reproducibility of the findings in a second iteration of the study. To quantify the possible impact of this potential for selection bias, we conducted a *P*-value to discern whether the demographic differences between groups were statistically significant. We learned that the only significant difference between groups related to age. All other differences were statistically insignificant. Lastly, the primary focus of the vlog was to deliver the health information in an engaging manner that would be well received. More attention should have been focused on delivering health education in a way that was clear and readily comprehensible.

### Future Research

As of April 2019, YouTube ranks as the social network site with the second highest internet traffic volume worldwide.^48^–^50^ Black millennials reported daily YouTube use.^50^ Gabarron and Wynn (2016) put forth a call to action, encouraging future researchers to conduct more in-depth studies using social media to promote sexual education to create evidence supporting the efficacy of this medium.^51^ There is potential for using vlogs for peer education, which has been established as one of the most effective intervention strategies,^36^ through a YouTube channel as an avenue to refute common misconceptions about the transmission of HIV/STIs. Importantly, HIV education interventions tend to increase opportunities for subsequent behavior changes that promote health and prevention of disease transmission when implemented together with behavioral change strategies.^36^,^52^ Given those findings, it is pertinent to develop and implement research that connects vlogs to strategies that motivate changes in behavior. Future research using vlogs may benefit from a larger sample size and additional adjusted analyses to contribute findings capable of reproducibility. Leveraging social media influences as behavioral scientists develop interventions is vital, now and in the future.

## CONCLUSION

Leveraging vlogs to test innovative ways of providing sexual health education in a culturally competent manner for Black women has not been readily adopted within clinical or public health practice. We used actors who reflected the target audience in terms of race, gender, and colloquialism as the communication method in an effort to enhance cultural relevance with the intervention’s content. This model can be built upon to design and implement innovative intervention strategies that connect with the audience in a way that aligns with societal and community-based communication norms.

## Figures and Tables

**Table 1:** Eligibility criteria of cisgender Black women.

Criteria	Description
Inclusion criteria	Cisgender women Race: Identified as Black or African American in the EHR Sexual orientation: Women who have sex with cisgender men Age: 18–45 years Reported sexual activity in the prior three months Presented to the ED with a non-emergent condition Had basic understanding of how to answer survey questions on a tablet device Had visual and comprehension capabilities
Exclusion criteria	Cisgender Black women <18 and >45 years of age Sexual orientation: sex with cisgender women Had a high acuity condition Had no technical competency on how to use a tablet device Had limited visual and comprehension capabilities

**Table 2:** Demographic description of the study population (N = 26 Black women).

Study arm	Taboo (N = 14)		Storytelling (N = 12)		P-value
			
Categories	N	Frequency (%)	N	Frequency (%)	
Race					0.88
African American	12	85.7	7	58.3	
Black	1	7.1	5	41.7	
Other^*^	1	7.1			
Gender at birth					
Female	14	100	12	100	
Age					0.05
18–19 years	0	0	5	16.7	
20–24 years	7	50.0	1	8.3	
25–29 years	3	21.4	3	25.0	
30–34 years	2	14.3	1	8.3	
35–40 years	0	0	4	33.3	
40–45 years	2	14.3	1	8.3	
Relationship status					0.70
Single	8	57.1	5	41.7	
In a relationship (not married)	2	14.3	4	33.3	
Living with partner (not married)	2	14.3	2	16.7	
Married	1	7.1	1	8.3	
Separated	1	7.1	0	0	
Highest level of education completed					0.74
Some secondary	3	21.4	2	16.7	
Completed secondary	5	35.7	4	33.3	
Some university	3	21.4	5	41.7	
Completed university	2	14.3	1	8.3	
Graduate/professional school	1	7.1	0	0	
Current employment status					0.66
Full-time (30–40 hours/week)	4	28.6	4	33.3	
Part-time (<30 hours/week)	4	28.6	2	16.7	
Occasional	1	7.1	0		
Unemployed	5	35.7	6	50.0	
Current monthly household income					0.36
< $500	2	14.3	4	33.3	
$501–$1,000	4	28.6	3	25.0	
$1,001 – $1,500	1	7.1	3	25.0	
$1,501–$2,000	2	14.3	0	0	
$2,001 – $2,500	1	7.1	1	8.3	
≥ $3,001 and over	4	28.6	1	8.3	

**Table 3:** Description of reported behaviors among the study cohort.

Study Arm	Taboo		Storytelling		P-value
			
Categories	N= 14	%	N= 12	%	
Heterosexual encounter in the prior 3 months					0.91
Yes	13	92.9	11	91.7	
No	1	7.1	1	8.3	
Missing					
Condomless sex in the prior 3 months					0.91
Yes	13	92.9	11	91.7	
Missing	1	7.1	1	8.3	
Knowledge of current HIV status					0.39
Yes	11	78.3	11	91.7	
No	3	21.4	1	8.3	
Missing					
If yes, what is your current HIV status?					
Negative	10	71.4	11	91.7	
Positive	1	7.1	0	0	
Missing	3	21.4	1	8.3	
History of an STI					0.23
Yes	8	57.1	4	33.3	
No	6	42.9	8	66.7	
Access to social media					NC
Yes	14	100	12	100	
No	0	0	0	0	

*HIV,* human immunodeficiency virus; *STI,* sexually transmitted infection; *NC,* not computed due to lack of variance in responses across groups.

**Table 4:** An evaluation of changes in knowledge between the pre- and post-intervention assessments.

Intervention strategy	Areas	Taboo (N = 14)					Storytelling (N = 12)				
		
True (T) / false (F) statement (correct response)			Pre-test	Post-test	X^2^ test	P-value	2-sided	Pre test	Post test	X^2^ test	P-value
			Knowledge increased								
If I am taking birth control, then I cannot get an STI. (F)	1	True	1	0	1.04	.31	True	-	-	-	-
		False	13	14			False	-	-	-	-
If my partner does not cum inside me, then I cannot get an STI. (F)	2	True	-	-	-	-	True	1	0	NC	
		False	-	-	-	-	False	11	12		
There is no risk of getting an STI if you urinate after having sex. (F)	3	True	-	-	-	-	True	3	1	1.20	.27
		False	-	-	-	-	False	9	11		
			No intervention effect								
If my partner does not cum inside me, then I cannot get an STI. (F)	2	True	14	14	NC		True	-	-	-	-
		False	0	0			False	-	-	-	-
There is no risk of getting an STI if you urinate after having sex. (F)	3	True	1	1	.00	1.000	True	-	-	-	-
		False	13	13			False	-	-	-	-
If I have only had sex with one person, then I cannot have an STI. (F)	4	True	0	0	NC		True	0	0	NC	
		False	14	14			False	12	12		
			Knowledge decreased								
If I am taking birth control, then I cannot get an STI. (F)	1	True	-	-	-	-	True	0	2	2.18	.14
		False	-	-	-	-	False	12	10		
If someone has HIV or an STI, I would be able to tell. (F)	5	True	1	13	20.57	.000	True	1	9		
		False	13	1			False	11	3	10.97	.001
The risk of getting a new STI is higher if I already have an STI. (T)	6	True	9	0	13.26	.000	True	9	2	8.22	.004
		False	5	14			False	3	10		
HIV and STIs can be spread through shaking hands, touching doorknobs, and sitting on toilet seats. (F)	7	True	3	13	14.58	.000	True	1	11	14.06	.000
		False	11	1			False	11	1		
Chlamydia and gonorrhea are two STI that can be treated and cured using medication. (T)	8	True	13	0	24.27	.000	True	12	1	20.31	.000
		False	1	14			False	0	11		
If my sexual partner and I both have HIV, then we do not need to use condoms. (F)	9	True	3	14	18.12	.000	True	1	11	20.33	.000
		False	11	0			False	11	0		
It is possible to have an STI without feeling sick. (T)	10	True	13	2	17.37	.000	True	12	3	14.40	.000
		False	1	12			False	0	9		
Baby oil and Vaseline are good to use with a latex condom as lube. (F)	11	True	2	14	21.00	.000	True	2	12	17.14	.000
		False	12	0			False	10	0		
Condoms should be used with anal sex. (T)	12	True	12	1	19.58	.000	True	12	1	20.31	.000
		False	1	13			False	0	11		
Every STI has a cure. (F)	13	True	1	4	2.19	.139	True	1	3	1.20	.273
		False	13	10			False	11	9		
Although there is no cure for HIV, it is possible to live a long, healthy life with the help of proper medications. (T)	14	True	14	13	1.04	.309	True	12	11	1.04	.307
		False	0	1			False	0	1		
If I am HIV positive, I cannot spread HIV to my sexual partner if I am taking the right medicine. (T)	15	True	6	0	7.64	.006	True	4	0	4.80	.028
		False	8	14			False	8	12		

STI, sexually transmitted infection; HIV, human immunodeficiency virus; NC, not computed because the question is a constant.
